# Iatrogenic hypervitaminosis D as an unusual cause of persistent vomiting: a case report

**DOI:** 10.1186/1752-1947-8-74

**Published:** 2014-02-26

**Authors:** Rinkesh Kumar Bansal, Pankaj Tyagi, Praveen Sharma, Vikas Singla, Veronica Arora, Naresh Bansal, Ashish Kumar, Anil Arora

**Affiliations:** 1 Department of Gastroenterology & Hepatology, Sir Ganga Ram Hospital, Rajinder Nagar, New Delhi 110 060, India

**Keywords:** Hypercalcemia, Hypervitaminosis, Vitamin D

## Abstract

**Introduction:**

Vitamin D is increasingly recognized to have several beneficial effects. Vitamin D deficiency is widely prevalent. Physicians often treat patients with high doses of vitamin D for various ailments without any monitoring for adverse effects and the prescribed doses often far exceed requirements resulting in toxicity. We present here a classic case of iatrogenic hypervitaminosis D, which presented with persistent vomiting and acute renal failure.

**Case presentation:**

Here we present a case of a 45-year-old Asian Indian woman who presented to us with persistent vomiting the cause of which was iatrogenic hypervitaminosis D. She was treated with intravenous fluid, diuretics and calcitonin and had clinical improvement.

**Conclusions:**

We suggest that in any patient presenting with persistent vomiting and hypercalcemia, particularly in the presence of normal parathyroid hormone, a diagnosis of overdose of vitamin D should be suspected. Its treatment not only alleviates symptoms but also prevents ongoing acute kidney injury.

## Introduction

Vitamin D is an important prohormone which plays an important role in calcium homeostasis and bone mineral metabolism. Vitamin D also subserves in a wide range of fundamental biological functions like cell differentiation, inhibition of cell growth as well as immune modulation. In adults, vitamin D deficiency leads to a mineralization defect in the skeleton causing osteomalacia. In addition, the secondary hyperparathyroidism associated with vitamin D deficiency enhances mobilization of calcium from the skeleton, resulting in porotic bone and increasing fracture risk. Although no minimum daily dietary intake of vitamin D has been identified, for adults exposed to ample sunlight less than 2.0μg/day (that is 80 units/day) of dietary intake is associated with its overt deficiency in adults. The US National Academy of Science sets a recommended daily allowance for vitamin D as 15μg/day (that is 600 units/day) for people below 70 years, and for people older than 70 years as 20μg/day (that is 800 units/day). Sun exposure and use of fortified or enriched foods are the methods by which mild vitamin D deficiency can be corrected. Moderate to severe vitamin D deficiency can be treated by oral administration of pharmacological dose of vitamin D 50,000IU/week for 6 to 8 weeks [1]. Due to a wide therapeutic index, vitamin D toxicity is extremely rare; however, it does occur at excessively high doses.

We present here a classic case of iatrogenic hypervitaminosis D, which presented with persistent vomiting and acute renal failure.

## Case presentation

A 45-year-old Asian Indian woman was admitted to our hospital with a history of recurrent vomiting, pain in her abdomen, polydipsia, anorexia and constipation for 1.5 months. There was no history of fever, altered sensorium, headache, cough, shortness of breath, or urinary symptoms. She had been hypertensive for the past year; 2 months ago she underwent arthroscopic repair of her right knee cruciate ligament.

On physical examination, she was conscious and oriented. Pallor was present; icterus and edema were absent. Her vitals were: blood pressure 140/100mmHg, pulse 84/minute, respiratory rate 20/minute, and temperature 37.6°C. An abdominal examination revealed no abnormalities. The results of her chest and cardiovascular examinations were also normal. An examination of her central nervous system showed generalized muscle weakness without any focal neurological deficit. Her deep tendon reflexes were normal.

On laboratory investigation the following results were obtained: hemoglobin 9.2g/dL, white blood cell count 12.2×10^3^/mm^3^, platelet count 242×10^3^/mL, blood urea nitrogen (BUN) 57mg/dL, creatinine 4.1mg/dL, sodium 139mEq/L, potassium 3.7mEq/L, serum calcium 11.54mEq/L, phosphorous 2.8mEq/L, serum bilirubin 0.60mg/dL, protein 6.3g/dL, albumin 3.6g/dL, globulin 2.7g/dL, alanine transaminase 22IU/L, aspartate transaminase 20IU/L, alkaline phosphatase 22IU/L, and erythrocyte sedimentation rate 76mm. The result of her Mantoux test was negative, her angiotensin-converting enzyme was 18.20mg/dL and her parathyroid hormone was 11.60pg/mL. The reference ranges of our lab for analytes are given in Table 1. Her urine analysis and microscopy were normal. Her urine for Bence Jones protein was negative. Protein electrophoresis did not reveal any monoclonal bands. Her chest X-ray was normal and her electrocardiogram showed low-voltage complexes. An ultrasound of her abdomen was normal. An ultrasound of her neck did not reveal any parathyroid mass. High-resolution computed tomography of her chest and magnetic resonance imaging of her brain were also essentially normal.

**Table 1:** Reference ranges for analytes

	
Hb	13–15g/dL
WBC	4000–10,000 per mm^3^
Platelet count	150–400×10^3^/mL
BUN	5–23mg/dL
Creatinine	0.6–1.3mg/dL
Sodium	132–148mEq/L
Potassium	3.5–5.5mEq/L
Calcium	8.2–10.4mg/dL
Phosphorous	2.8mg/dL
Serum bilirubin	0.2–1mg/dL
Protein	6.6–8.7g/dL
Albumin	3.5–5g/dL
Globulin	1.5–3.5g/dL
ALT	0–60IU/L
AST	0–40IU/L
ALP	39–117IU/L
ESR	0–10mm after end of first hour
ACE	<40mg/dL
PTH	12–64pg/mL

Abbreviations: ACE, angiotensin-converting enzyme; ALP, alkaline phosphatase; ALT, alanine transaminase; AST, aspartate transaminase; BUN, blood urea nitrogen; ESR, erythrocyte sedimentation rate; Hb, hemoglobin; PTH, parathyroid hormone; WBC, white blood cell count.

Hence the biochemical evaluation revealed only raised BUN and serum creatinine suggesting acute renal failure with hypercalcemia, in the absence of evidence of chronic kidney disease, multiple myeloma or hyperparathyroidism. A detailed history of previous treatment revealed that she had received an injection of Arachitol (vitamin D3) 600,000IU intramuscularly, every alternate day for 10 doses just after her knee surgery 2 months back. We found her serum 25-hydroxyvitamin D (25(OH)D) level to be 150ng/mL which was in the toxic range (normal 20 to 30ng/mL). So a diagnosis of vitamin D toxicity-induced acute kidney injury and hypercalcemia was established. To rule out chronic kidney disease a nephrology consultation was sought and ultrasound for her kidneys and routine urine microscopy were ordered, which were normal.

For hypercalcemia, she was treated with intravenous fluid, diuretics and calcitonin and had clinical improvement. Her serum calcium and creatinine levels were monitored regularly; they gradually declined to normal levels in the next 15 days. She was discharged with a prescription of an antihypertensive and calcium-restricted diet along with good hydration; she is doing well.

## Discussion

In this case report a classic case of iatrogenic hypervitaminosis D, which presented with persistent vomiting and acute renal failure, has been described. Hypervitaminosis D can occur when pharmaceutical vitamin D is taken in excess, as in our case. The manifestation could be related to hypercalcemia, or acute kidney injury, or both as in our case [2].

The biologically active metabolite of vitamin D, 1,25-dihydroxyvitamin D(3), affects mineral homeostasis and has numerous other diverse physiologic functions including effects on growth of cancer cells and protection against some immune disorders [3]. Adequate vitamin D status has been linked to decreased risks of developing specific cancers, including cancers of the esophagus, stomach, colon, rectum, gallbladder, pancreas, lung, breast, uterus, ovary, prostate, urinary bladder, kidney, skin, thyroid, and hematopoietic system (for example, Hodgkin’s lymphoma, non-Hodgkin’s lymphoma, multiple myeloma); bacterial infections; rheumatoid arthritis; Crohn’s disease; periodontal disease; multiple sclerosis; asthma; type 2 diabetes; cardiovascular disease; stroke; peripheral artery disease; hypertension; chronic kidney disease; muscle weakness; cognitive impairment; Alzheimer’s disease; clinical depression; and premature death [4]. Whereas inadequate vitamin D status during human pregnancy may be associated with increased risk for the development of type 1 diabetes in the offspring [4].

Vitamin D deficiency can be treated by oral administration of a pharmacological dose of vitamin D 50,000IU/week for 6 to 8 weeks [1]. Even though vitamin D toxicity is extremely rare, due to the wide therapeutic index, it does occur at excessively high doses. The guidelines of the Food and Nutrition Board of the USA specify 2000IU as the highest vitamin D intake that healthy adults can consume daily without risk of hypercalcemia [5]. However, these patients should be monitored by periodic estimation of 24-hour urinary calcium excretion, which should not exceed 250mg. Very little is known about the mechanism of vitamin D toxicity [6]. The lipophilic nature of vitamin D explains its adipose tissue distribution and its slow turnover in the body (half-life approximately 2 months), whereas its main transported metabolite, 25-hydroxyvitamin D(3) (25(OH)D(3)), has a half-life of approximately 15 days. Animal experiments involving vitamin D3 intoxication have established that 25(OH)D(3) can reach concentrations up to 2.5micromol/L, at which it is accompanied by hypercalcemia and other pathological sequelae resulting from a high calcium/phosphate product [6]. Although current data support the viewpoint that the biomarker plasma 25(OH)D concentration must rise above 750nmol/L to produce vitamin D toxicity, the more prudent upper limit of 250nmol/L might be retained to ensure a wide safety margin. There also is evidence that despite the current heavy reliance on serum 25(OH)D(3) concentration for the diagnosis of an individual’s vitamin D status, local tissue vitamin D intoxication may be present in individuals with much lower serum 25(OH)D(3) concentrations than are currently appreciated [6]. Only rarely are the symptoms of local tissue vitamin D intoxication associated with vitamin D status or intake. An individual’s serum 25(OH)D(3) concentration may appear to be “low” for reasons totally independent of sunlight exposure or vitamin D intake. Serum 25(OH)D(3) concentration is only poorly responsive to increases in vitamin D intake, and the prolonged routine consumption of thousands of international units of vitamin D may interfere with the regulation of phosphate homeostasis by fibroblast growth factor-23 and the Klotho gene product, with consequences that are detrimental to human health [6].

Clinical manifestations of vitamin D toxicity include: hypercalcemia, hypercalciuria, kidney stones, hyperphosphatemia, polyuria, polydipsia, ectopic calcification of soft tissues (kidney and lung), nausea, vomiting, anorexia, constipation, headache, and hypertension [7,8]. In a recent paper Pandita *et al.*[9] reported a case series of 15 patients (most of them elderly) with iatrogenic symptomatic hypercalcemia in whom toxicity occurred due to empirical excessive administration of vitamin D by oral and parenteral route. To prevent iatrogenic vitamin D toxicity the authors suggested that awareness should be increased among healthcare providers regarding the toxic potential of mega doses of vitamin D, despite its wide margin of safety [9]. In another case series by Koul *et al.*[10] 10 cases of hypercalcemia due to vitamin D intoxication were reported with features of vomiting, polyuria, polydipsia, encephalopathy and renal dysfunction. All the patients had demonstrable hypercalcemia and vitamin D levels were high in nine of the 10 cases. The patients had received high doses of vitamin D and no other cause of hypercalcemia was identified. Treatment of hypercalcemia resulted in clinical recovery in nine cases [10].

## Conclusions

Thus we conclude that if a patient presents with persistent vomiting and hypercalcemia particularly in the presence of normal parathyroid hormone, then a diagnosis of overdose of vitamin D should be suspected because its correction not only alleviates symptoms but can also prevent acute kidney injury.

## Consent

Written informed consent was obtained from the patient for publication of this manuscript and accompanying images. A copy of the written consent is available for review by the Editor-in-Chief of this journal.

## Abbreviations

25(OH)D: 25-hydroxyvitamin D; 25(OH)D(3): 25-hydroxyvitamin D(3); BUN: Blood urea nitrogen.

## Competing interests

The authors declare that they have no competing interests.

## Authors’ contributions

RKB was the resident in charge of the case and was a major contributor in writing the initial draft of the manuscript. PT, PS, VS, NB, VA and AK contributed in writing and editing the manuscript. NB was the attending physician in charge of the case and was responsible for sections on the diagnosis and management of the patient. AA was the unit head and was responsible for management of the patient and supervised and edited the case report. All authors read and approved the final manuscript.
